# Metamorphosis-related changes in the free fatty acid profiles of *Sarcophaga *(*Liopygia*)* argyrostoma* (Robineau-Desvoidy, 1830)

**DOI:** 10.1038/s41598-020-74475-1

**Published:** 2020-10-15

**Authors:** Agata Kaczmarek, Anna Katarzyna Wrońska, Michalina Kazek, Mieczysława Irena Boguś

**Affiliations:** 1 The Witold Stefański Institute of Parasitology, Polish Academy of Sciences, Warsaw, Poland; 2 Biomibo, Warsaw, Poland

**Keywords:** Entomology, Fatty acids

## Abstract

The flies of the Sarcophagidae, widespread throughout the temperate zone, are of great significance in Medicine, Veterinary science, Forensics and Entomotoxicology. Lipids are important elements of cell and organelle membranes and a source of energy for embryogenesis, metamorphosis and flight. Cuticular lipids protect from desiccation and act as recognition cues for species, nest mates and castes, and are a source of various pheromones. The free fatty acid (FFA) profile of cuticular and internal extracts of *Sarcophaga *(*Liopygia*)* argyrostoma* (Robineau-Desvoidy, 1830) larvae, pupae and adults was determined by gas chromatography–mass spectrometry (GC–MS). The larvae, pupae and adults contained FFAs from C5:0 to C28:0. The extracts differed quantitatively and qualitatively from each other: C18:1 > C16:1 > C16:0 > C18:0 predominated in the cuticular and internal extracts from the larvae and adults, while 18:1 > C16:0 > C16:1 > C18:0 predominated in the pupae. The FFA profile of the cuticle varies considerably between each development stage: C23:0 and C25:0 are only present in larvae, C28:0 in the pupal cuticle, and C12:1 and C18:3 in internal extracts from adults. The mechanisms underlying this diversity are discussed herein.

## Introduction

The Sarcophagidae is a large family represented by more than 3000 species^1^ and its subfamily Sarcophaginae has the highest diversity of species in Central Europe^2^. Its members present a variety of feeding habits, including sarcophagy, necrophagy and/or coprophagy, especially in the larval stage, and therefore play an important role in matter decomposition^3–5^. Sarcophaga flies are natural hosts of the parasitic wasp *Nasonia vitripennis* and are often used in laboratory studies for wasp culture^6–8^. They also have medical and veterinary significance as obligatory and facultative parasitoids, predators, and myiasis-causing factors^9–16^. Several species are synanthropic and may be responsible for the mechanical transmission of pathogens to food and humans, with potential consequences for public health^12,17,18^. They are suitable animals for studying the physiology and biochemistry of insects, particularly their endocrinology^19,20^, diapause^21–23^, reproduction^24^ and immunity^25–27^. In addition, they are regarded as having high forensic value, due to their ovoviviparity (or ovolarviparity): they deposit maggots instead of eggs directly on a corpse, and due to the larger size of the larvae and their higher efficiency of sarcophagid flies, they act as convenient markers of decay^28–31^. Additionally, drugs or other toxic substances that could be undetected in decomposed tissues can be detected in the tissues of the larvae found in the corpse (entomotoxicology)^28,32^. However, their use in criminal investigations is still limited due to difficulties in species identification. Such difficulties are present for almost all life stages, especially the larval stages, which demonstrate little morphological diversity; there is also a need to better understand the ecology, behaviour and distribution of the insects, and to improve sample collection, as the larvae can spread for up to 10 m from the cadaver^31,33–36^.

Due to their high medical, veterinary and forensic importance, there is a great need to better understand the morphological and physiological processes of *Sarcophaga* flies. The present study examines the changes in lipid profile during three development stages of *Sarcophaga (Liopygia) argyrostoma* (Robineau-Desvoidy, 1830). It has often been recorded that FFA profiles differ considerably between development stages, even within a single species^37–44^.

Lipids are important components of cells and play a key role in the well-being of insects. Internal free fatty acids (FFAs) are essential parts of cell and organelle membranes, serve as important sources of energy, and act as precursors for secondary metabolites, waxes, pheromones and defensive secretions^45–47^.

Cuticular lipids are a diverse group of compounds, whose content and composition in the insect vary according to diet and climate. Cuticular lipids (including FFAs) perform many important functions associated with maintaining homeostasis within the insect. Most importantly, their presence minimizes transpiration and protects terrestrial insects from desiccation^48,49^. Cuticular lipids also play roles in several biochemical, physiological, and semiochemical (behaviour and signalling) processes. They act as recognition cues for species, nest mate and caste; they also serve as a reservoir for a suite of pheromones responsible for sexual attraction, epideictic activity (insect display behaviour), territorial markers, alarm, chemical defence, and thermoregulation, predator–prey and parasitoid-host interactions, and mimicry and camouflage^50–53^. The lipid profile of the insect cuticle is also a crucial indicator of susceptibility or resistance to fungal invasion^41,54,55^, and hence an understanding of its FFA profile could play a significant role in the identification of flies and the control of their populations. In addition, changes in the lipid composition related to the individual stages of development of holometabolous insects are also associated with differences in such aspects as body composition, lifestyle, diet and environment.

Hence, there is a great need for further research on the FFA profiles for insects. Therefore, the aim of this study was to describe metamorphosis-related changes in FFA profiles in the flesh fly *S. argyrostoma*.

## Results

The present work characterises the chemical composition of cuticular and internal FFAs of *S. argyrostoma*. Three types of extraction were performed for the larval, pupal and adult material. The cuticular lipids were found in the petroleum ether (extract I) and dichloromethane (extract II) extracts, and the internal lipids in extract III. The total masses of the extracts are shown in Table 1. Cuticular extracts of larvae amounted to 0.002 mg in the cuticular extracts (0.00017 mg per larva) and 0.900 mg in the internal extracts (0.075 mg per larva). Greater quantities of cuticular extracts were obtained from pupae 1.530 mg (0.077 mg per pupa) than larvae; however, lower quantities of internal extracts were obtained from pupae (0.520 mg; 0.026 mg per individual). The most efficient extraction was observed for adults: the cuticular extract yielded 5.630 mg (0.469 mg per insect), and the internal extract yielded 7.660 mg (0.638 mg per insect). The masses of the extracts are shown in Table 1.

**Table 1:** The numbers of *Sarcophaga argyrostoma* used and masses of extracts obtained.

Extracts made from	N	Insects mass (g)	Extract mass					
			(mg)			(mg/insect)		
			I	II	III	I	II	III
Larvae	12	1.039	0.001	0.001	0.900	0.0001	0.0001	0.075
Pupae	20	0.972	0.600	0.930	0.520	0.030	0.047	0.026
Adults	12	1.071	2.060	3.570	7.660	0.172	0.298	0.638

*N* total number of individuals, *I* petroleum ether extract, *II* dichloromethane extract, *III* dichloromethane extract after sonication.

These extracts were further analysed by GC–MS. A comparison of the FFA profiles of the cuticle surface (sum of extracts I and II) and the internal structures of the insect is given in Table 2; the raw data is appended in Table S1. The highest total FFA content was observed in adults, in both the cuticular (1865.278 ± 19.580 µg/g of insect body) and internal extracts (3811.660 ± 9.217 µg/g of insect body), while the lowest was observed for pupae: 70.821 ± 2.381 µg/g insect body mass in the cuticular extract, and 63.654 ± 1.167 µg/g insect body mass in the internal extract. In larvae, the total FFA content equalled 186.576 ± 7.550 μg/g insect body mass in the cuticular extracts, and 190.665 ± 8.849 μg/g insect body mass in the internal extract. Only the extracts from the adults demonstrated a statistically significant difference in total FFA content between cuticular and internal fractions (p < 0.001).

**Table 2:** Fatty acid contents in the cuticular and internal lipids extracted from Sarcophaga argyrostoma [µg/g of body mass ± SD].

FFA	Larvae		Pupae		Adults	
	Cuticular	Internal	Cuticular	Internal	Cuticular	Internal
C5:0	0.147 ± 0.008^A–C^	ND^A^	0.021 ± 0.004^B^	0.013 ± 0.005^C^	0.732 ± 0.019^A–C^	0.212 ± 0.026^A–C^
C6:0	0.234 ± 0.001^A^	0.349 ± 0.053^B^	0.470 ± 0.016^C^	0.224 ± 0.018^D^	1.785 ± 0.155^A–D^	1.147 ± 0.096^A–D^
C7:0	0.056 ± 0.000^A^	0.057 ± 0.003^B^	0.031 ± 0.001^C^	0.034 ± 0.002^D^	0.636 ± 0.131^A–D^	0.308 ± 0.020^A–D^
C8:0	0.400 ± 0.007^A^	0.143 ± 0.007^B^	0.195 ± 0.032^C^	0.059 ± 0.006^D^	1.091 ± 0.288^A–E^	0.386 ± 0.052^E^
C9:0	0.824 ± 0.000^AB^	0.451 ± 0.010^A^	0.351 ± 0.018^B^	0.183 ± 0.014^AB^	1.951 ± 0.073^AB^	1.146 ± 0.076^AB^
C10:0	0.108 ± 0.003^A^	0.048 ± 0.002^B^	0.063 ± 0.003^C^	0.027 ± 0.005^D^	0.372 ± 0.042^A–D^	0.270 ± 0.045^A–D^
C11:0	0.043 ± 0.001^A^	0.036 ± 0.002^B^	ND^C^	ND^D^	2.512 ± 0.120^A–D^	3.122 ± 0.151^A–D^
C12:1	ND^A^	ND^B^	ND^C^	ND^D^	ND^E^	0.917 ± 0.049^A–E^
C12:0	0.493 ± 0.007^A^	0.357 ± 0.011^B^	0.216 ± 0.011^A^	0.094 ± 0.010^AB^	3.122 ± 0.149^AB^	2.791 ± 0.114^AB^
C13:0	0.049 ± 0.001^A^	0.020 ± 0.004^B^	0.027 ± 0.013^C^	ND^D^	0.225 ± 0.018^A–D^	0.269 ± 0.044^A–D^
C14:1	1.682 ± 0.033^A^	1.947 ± 0.063^B^	0.106 ± 0.016^A^	0.104 ± 0.014^B^	14.969 ± 0.522^AB^	21.864 ± 0.906^AB^
C14:0	3.843 ± 0.016^A^	4.257 ± 0.075^B^	1.498 ± 0.068^B^	0.754 ± 0.012^B^	25.098 ± 0.555^AB^	41.213 ± 1.096^AB^
C15:1	0.275 ± 0.012^A^	0.291 ± 0.032^B^	0.039 ± 0.007^A^	0.017 ± 0.005^B^	2.408 ± 0.055^AB^	4.091 ± 0.081^AB^
C15:0	0.533 ± 0.011^A^	0.473 ± 0.012^B^	0.299 ± 0.017^C^	0.193 ± 0.0013^D^	5.429 ± 0.151^A–D^	9.938 ± 0.287^A–D^
C16:1	41.580 ± 1.484^A^	55.917 ± 3.394^B^	8.454 ± 0.480^A^	9.884 ± 0.169^B^	476.463 ± 17.290^AB^	916.970 ± 4.513^AB^
C16:0	33.788 ± 0.019^A^	33.230 ± 0.473^B^	15.492 ± 0.528^A^	11.698 ± 0.173^B^	311.453 ± 0.891^AB^	640.327 ± 5.246^AB^
C17:1	1.242 ± 0.218^A^	1.938 ± 0.004^B^	0.362 ± 0.022^C^	0.258 ± 0.005^D^	26.128 ± 0.321^A–D^	48.126 ± 4.842^A–D^
C17:0	0.810 ± 0.008^A–C^	0.425 ± 0.008^A^	0.454 ± 0.025^B^	0.269 ± 0.035^C^	5.915 ± 0.137^A–C^	11.704 ± 0.090^A–C^
C18:3	ND^A^	ND^B^	ND^C^	ND^D^	ND^E^	14.205 ± 0.476^A–E^
C18:2	1.901 ± 0.132^A^	1.519 ± 0.194^B^	1.005 ± 0.018^C^	2.957 ± 0.299^D^	12.575 ± 0.172^A–D^	25.414 ± 2.157^A–D^
C18:1	77.238 ± 5.622^A^	77.397 ± 10.728^B^	31.081 ± 0.969^C^	30.459 ± 0.279^D^	891.201 ± 35.577^A–D^	1872.927 ± 8.787^A–D^
C18:0	12.296 ± 0.109^AB^	6.203 ± 0.081^A^	6.188 ± 0.184^B^	2.987 ± 0.071^AB^	32.122 ± 1.073^AB^	67.515 ± 1.235^AB^
C19:1	0.215 ± 0.012^AB^	0.119 ± 0.005^C^	ND^AC^	ND^BC^	1.647 ± 0.045^AC^	3.771 ± 0.055^AC^
C19:0	0.155 ± 0.004^A^	0.063 ± 0.003^B^	ND^C^	ND^D^	0.266 ± 0.056^CD^	0.731 ± 0.143^A–D^
C20:5	0.512 ± 0.006^A^	0.746 ± 0.010^B^	ND^C^	0.744 ± 0.034^D^	17.893 ± 0.345^A–D^	60.680 ± 0.503^A–D^
C20:4	1.427 ± 0.016^A^	1.981 ± 0.005^C^	ND^C^	1.237 ± 0.048^C^	14.131 ± 0.070^A–C^	42.805 ± 0.567^A–C^
C20:3	0.231 ± 0.022^A^	0.255 ± 0.019^B^	0.228 ± 0.017^C^	ND^D^	2.370 ± 0.749^A–D^	3.802 ± 0.036^A–D^
C20:1	0.923 ± 0.057^A^	0.296 ± 0.017^B^	0.349 ± 0.022^C^	ND^D^	2.091 ± 0.287^A–D^	4.262 ± 0.730^A–D^
C20:0	0.488 ± 0.005^A^	0.278 ± 0.020^B^	0.700 ± 0.012^C^	0.277 ± 0.028^D^	2.569 ± 0.423^A–D^	4.995 ± 0.523^A–D^
C22:0	1.348 ± 0.014^ABD^	0.492 ± 0.005^ACD^	1.138 ± 0.012^CF^	0.548 ± 0.053^BF^	2.541 ± 0.236^AF^	2.443 ± 0.058^DF^
C23:0	0.270 ± 0.001^A–D^	0.160 ± 0.026^A–D^	ND^A^	ND^B^	ND^C^	ND^D^
C24:0	1.910 ± 0.061^A–D^	0.684 ± 0.007^AD^	1.178 ± 0.044^BF^	0.633 ± 0.058^CE^	3.202 ± 0.105^A–C^	3.312 ± 0.433^D–F^
C25:0	0.214 ± 0.008^A–D^	0.045 ± 0.004^A–D^	ND^A^	ND^B^	ND^C^	ND^D^
C26:0	1.342 ± 0.016^AB^	0.489 ± 0.007^AB^	0.877 ± 0.027^AB^	ND^A^	2.385 ± 0.198^AB^	ND^B^
C28:0	ND^A^	ND^B^	0.747 ± 0.100^A–E^	ND^C^	ND^D^	ND^E^
Sum of FFA	186.576 ± 7.550^AB^	190.665 ± 8.849^CD^	70.821 ± 2.381^AC^	63.654 ± 1.167^BD^	1865.278 ± 19.580^A–D^	3811.660 ± 9.217^A–D^

*FFA* free fatty acids, *SD* standard deviation, *ND* not detected; statistically significant differences are marked with the same letters (ANOVA, Test HSD Tukey, p < 0.05), see Table S1 for raw data.

**Figure 1:**
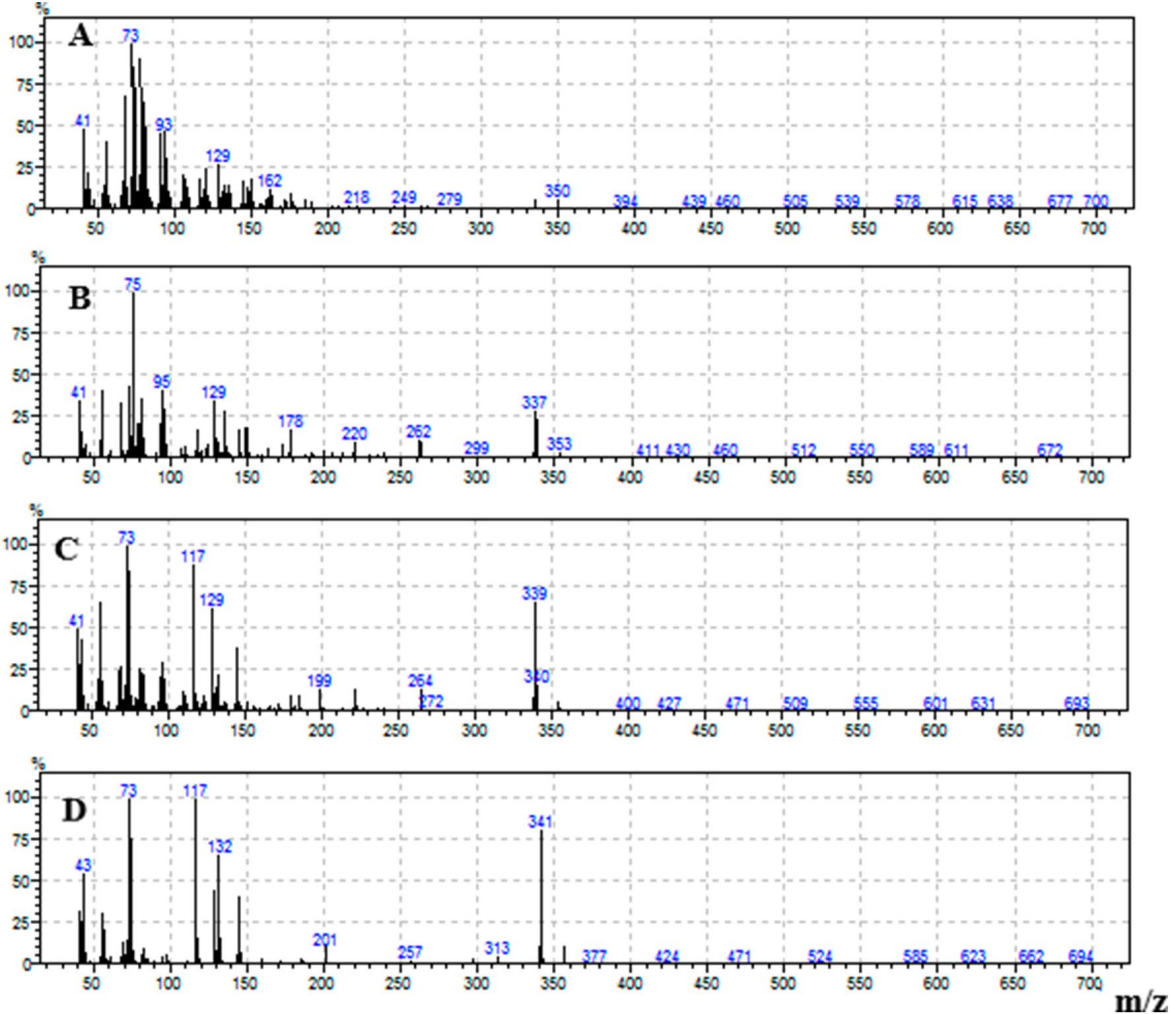
Mass spectra of the trimethylsilyl ester of octadecatrienoic acid, C18:3 (**A**), octadecadienoic acid C18:2 (**B**), octadecenoic acid C18:1 (**C**), octadecanoic acid C18:0 (**D**).

The individual FFAs present in each extract were identified and quantified. Example mass spectra of the trimethylsilyl (TMS) esters of octadecatrienoic acid (C18:3), octadecadienoic acid (C18:2), octadecenoic acid (C18:1) and octadecanoic acid (C18:0) are shown in Fig. 1.

The extracts from *S. argyrostoma* larvae were found to contain 33 FFAs (Table 2). The cuticular extracts contained 31 FFAs from C5:0 to C26:0: 21 saturated (C5:0–C20:0, C22:0–C26:0) and 11 unsaturated (C14:1, C15:1, C16:1, C17:1, C18:2, C18:1, C19:1, C20:5, C20:4, C20:3 and C20:1).

**Figure 2:**
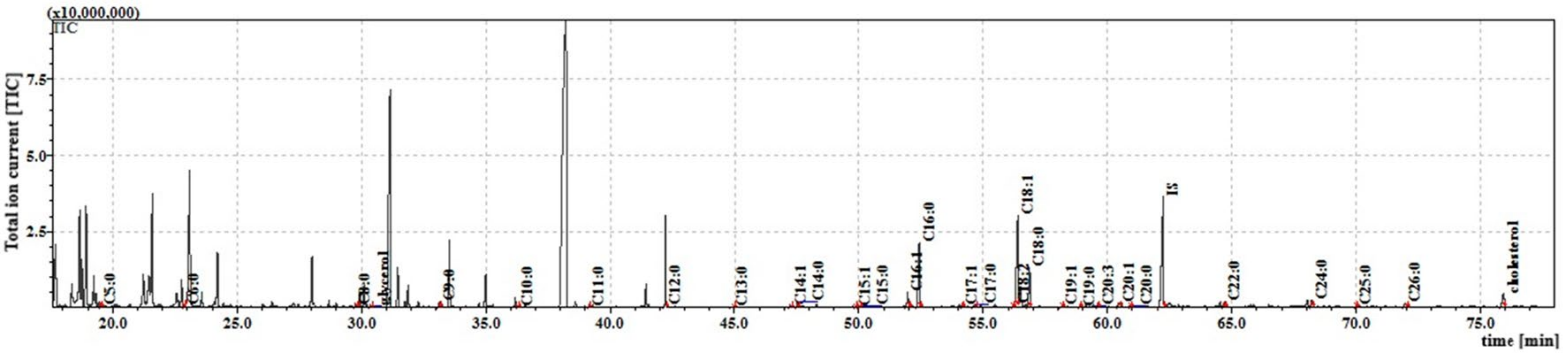
The total ion current (TIC) chromatogram of fatty acids (TMS esters) of the ether extract (Extract I) from *S. argyrostoma* larvae. Internal standard (IS, 19-methylarachidic acid); fatty acids and molecular ions: pentanoic acid (C5:0, m/ z = 174), hexanoic acid (C6:0, m/z = 188), octanoic acid (C8:0, m/z = 216), nonanoic acid (C9:0, m/z = 230), decanoic acid (C10:0, m/z = 244), undecanoic acid (C11:0, n/z = 258), dodecanoic acid (C12:0, m/z = 272), tridecanoic acid (C13:0, m/z = 286), tetradecenoic acid (C14:1, m/z = 298), tetradecanoic acid (C14:0, m/z = 300), pentadecenoic acid (C15:1, m/z = 312), pentadecanoic acid (C15:0, m/z = 314), hexadecenoic acid (C16:1, m/z = 326), hexadecanoic acid (C16:0, m/z = 328), heptadecenoic acid (C17:1, m/z = 340), heptadecanoic acid (C17:0, m/z = 342), octadecadienic acid (C18:2, m/z = 352), 17-octadecenoic acid (C18:1, m/z = 354), 18-octadecanoic acid (C18:0, m/z = 356), nonadecenoic acid (C19:1, m/z = 368), nonadecanoic acid (C19:0, m/z = 370), eicosatrienoic acid (C20:3, m/z = 378), eicosenoic acid (C20:1, m/z = 382), eicosanoic acid (C20:0, m/z = 384), docosanoic acid (C22:0, m/z = 412), etracosanoic acid (C24:0, m/z = 440), pentacosanoic acid (C25:0, m/z = 454), hexacosanoic acid (C26:0, m/z = 468).

The internal lipids had a similar fatty acid profile, with the exception that C5:0 was absent. Most FFAs were present at similar levels; however, C9:0, C17:0, C18:0, C22:0, C23:0, C24:0, C25:0 and C26:0 were significantly higher in the cuticle extract. The two extracts were found to have similar total amounts of FFAs. The total ion current (TIC) chromatogram of fatty acids (TMS esters) of the ether extract (Extract I) from the larvae is given in Fig. 2.

**Figure 3:**
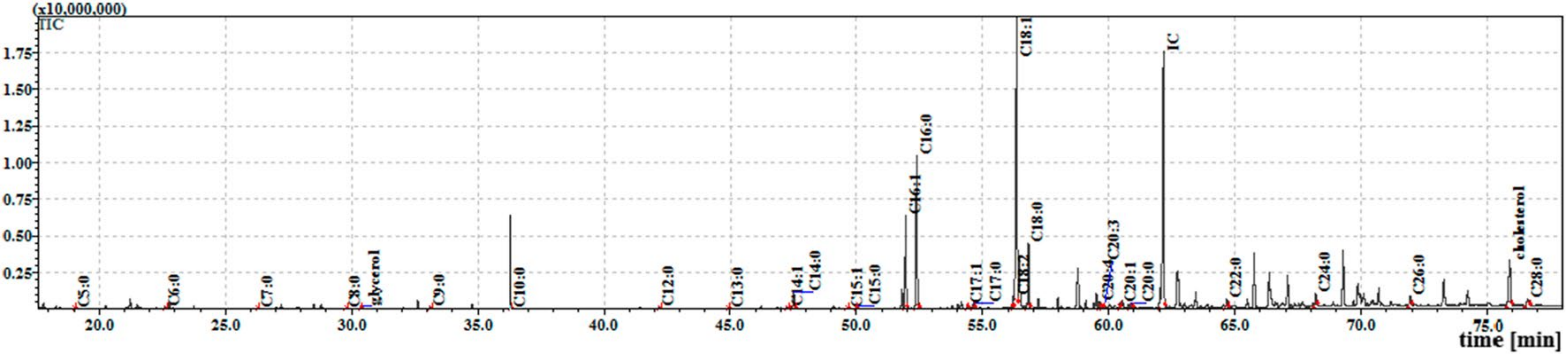
The total ion current (TIC) chromatogram of fatty acids (TMS esters) of the dichloromethane extract (Extract II) from *S. argyrostoma* pupae. Internal standard (IS, 19-methylarachidic acid); fatty acids and molecular ions: pentanoic acid (C5:0, m/ z = 174 hexanoic acid (C6:0, m/z = 188), heptanoic acid (C7:0, m/z = 202), octanoic acid (C8:0, m/z = 216), nonanoic acid (C9:0, m/z = 230), decanoic acid (C10:0, m/z = 244), dodecanoic acid (C12:0, m/z = 272), tridecanoic acid (C13:0, m/z = 286), tetradecenoic acid (C14:1, m/z = 298), tetradecanoic acid (C14:0, m/z = 300), pentadecanoic acid (C15:0, m/z = 314), hexadecenoic acid (C16:1, m/z = 326), hexadecanoic acid (C16:0, m/z = 328), heptadecenoic acid (C17:1, m/z = 340), heptadecanoic acid (C17:0, m/z = 342), octadecadienic acid (C18:2, m/z = 352), octadecenoic acid (C18:1, m/z = 354), octadecanoic acid (C18:0, m/z = 356), eicosatrienoic acid (C20:3, m/z = 378, eicosenoic acid (C20:1, m/z = 382), eicosanoic acid (C20:0, m/z = 384), docosanoic acid (C22:0, m/z = 412), tetracosanoic acid (C24:0, m/z = 440), hexacosanoic acid (C26:0, m/z = 468), octacosanoic acid (C28:0, m/z = 496).

The cuticular extracts from pupae contained 26 cuticular FFAs from C5:0 to C28:0: 18 saturated (C5:0–C10:0, C12:0–C18:0, C20:0, C22:0, C24:0, C26:0 and C28:0) and eight unsaturated (C14:1, C15:1, C16:1, C17:1, C18:2, C18:1, C20:3 and C20:1). The internal extract contained 23 FFAs. A comparison found the internal fractions to contain FFAs C20:4 and C20:5, absent in the cuticular fractions, but to lack C13:0, C20:3, C20:1, C26:0 and C28:0, present in the cuticular fractions. Most FFAs were found in similar concentrations in the cuticle and the internal extracts, except for C9:0, C12:0, C14:0, C18:0 and C22:0, which were significantly higher in the cuticle. The two extracts from the pupae also demonstrated similar total FFA levels to each other; however, these values were 2.6-times lower (cuticle) and three-times lower (internal) than the analogous extracts from the larvae. The TIC chromatogram of the TMS esters of the dichloromethane extract (Extract II) from *S. argyrostoma* pupae is given in Fig. 3.

**Figure 4:**
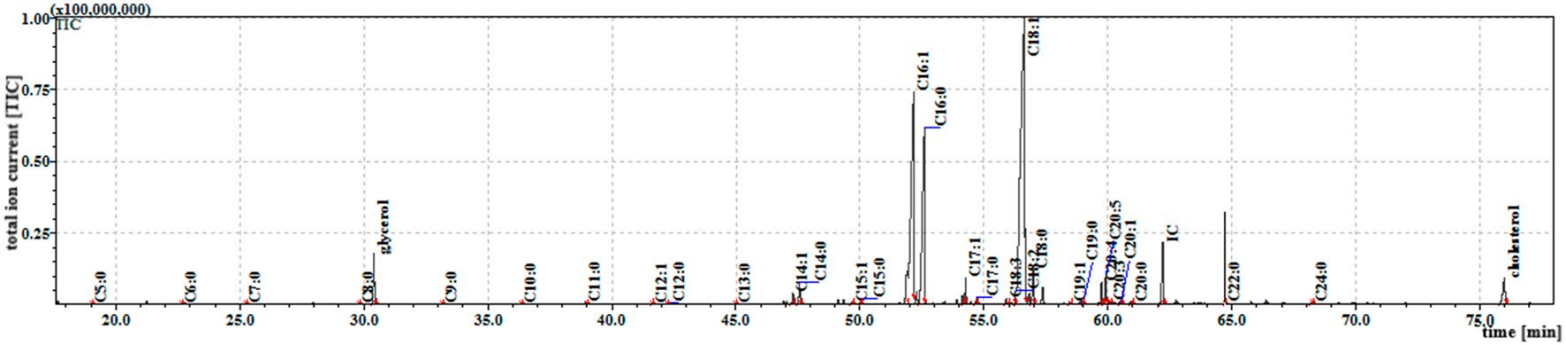
The total ion current (TIC) chromatogram of fatty acids (TMS esters) from sonicated from *S. argyrostoma* adults. Internal standard (IS, 19-methylarachidic acid); fatty acids and molecular ions: pentanoic acid (C5:0, m/ z = 174 hexanoic acid (C6:0, m/z = 188), heptanoic acid (C7:0, m/z = 202), octanoic acid (C8:0, m/z = 216), nonanoic acid (C9:0, m/z = 230), decanoic acid (C10:0, m/z = 244), undecanoic acid (C11:0, m/z = 258), dodecenoic acid (C12:1, m/z = 270), dodecanoic acid (C12:0, m/z = 272), tridecanoic acid (C13:0, m/z = 286), tetradecenoic acid (C14:1, m/z = 298), tetradecanoic acid (C14:0, m/z = 300), pentadecenoic acid (C15:1, m/z = 312), pentadecanoic acid (C15:0, m/z = 314), hexadecenoic acid (C16:1, m/z = 326), hexadecanoic acid (C16:0, m/z = 328), heptadecenoic acid (C17:1, m/z = 340), heptadecanoic acid (C17:0, m/z = 342), octadecatrienoic acid (C18:3, m/z = 350), octadecadienic acid (C18:2, m/z = 352), octadecenoic acid (C18:1, m/z = 354), octadecanoic acid (C18:0, m/z = 356), nonadecenoic acid (C19:1, m/z = 368), monadecanoic acid (C19:0, m/z = 370), eicosatetraenoic acid (C20:4, m/z = 376), eicosapentaenoic acid (C20:5, m/z = 374), eicosatrienoic acid (C20:3, m/z = 378, eicosenoic acid (C20:1, m/z = 382), eicosanoic acid (C20:0, m/z = 384), docosanoic acid (C22:0, m/z = 412), tetracosanoic acid (C24:0, m/z = 440).

Among the adults, the cuticular extracts contained 30 FFAs from C5:0 to C26:0: 19 saturated (C5:0–C20:0, C22:0, C24:0 and C26:0) and 11 unsaturated (C14:1, C15:1, C16:1, C17:1, C18:2, C18:1, C19:1, C20:5, C20:4, C20:3 and C20:1). A similar FFA profile was observed in the internal extracts; however, C26:0 was absent in extract III, and C12:1 and C18:3 were present, both of which were missing from the cuticular extracts. A higher concentration of short-chain FFAs was observed in the cuticular fraction, while the long-chain FFAs predominated in the internal fraction. However, the internal extracts demonstrated twice the total FFA content than the cuticular extracts (p < 0.001). The TIC chromatograms of TMS esters of the dichloromethane extract (Extract III) from the adults are given in Fig. 4.

The predominant FFA in all developmental stages was C18:1 (77 µg/g insect body mass in larvae, 30–31 µg/g insect body mass in pupae, and 891–1873 µg/g insect body mass in adults, respectively). High concentrations of C16:1 was measured only in larvae (42–56 µg/g of insect body) and adults (476–917 µg/g of insect body). The third most dominant acid was C16:0 (33–34 µg/g insect body mass in larvae, 12–15 µg/g insect body mass in pupae, and 311–640 µg/g insect body mass in adults).

Significant differences regarding the presence of individual FFAs were observed regarding the between developmental stages. C23:0 and C25:0 were observed only in larvae, whereas C28:0 was detected only in the cuticle of pupae. In turn, C12:1 and C18:3 were present only in internal extracts from adults. Interestingly, several FFAs present in larvae and adults were absent from pupae: C11:0, C19:1, and C19:0 absent from both extracts; C20:4 and C20:5 absent from the cuticle; C13:0, C20:3, C20:1, and C26:0 absent from the internal extract.

Glycerol and cholesterol were observed in all extracts. However, glycerol content was higher in the extract from the adults (23.366 ± 0.632 μg/g insect body mass in the cuticular extract and 93.437 ± 2.506 μg/g insect body mass in the internal extract) and larvae (3.064 ± 0.071 μg/g insect body mass in the cuticular extract and 30.370 ± 0.517 μg/g insect body mass in the internal extract) than from the pupae (0.316 ± 0.021 μg/g insect body mass in the cuticular extract and 0.3202 ± 0.014 μg/g insect body mass in the internal extract). In larvae and adults, the glycerol concentrations were ten-times and four-times higher in the internal extracts than the cuticle, while in pupae, both concentrations were nearly equal. The results have been showed in Table 3.

**Table 3:** Glycerol and cholesterol contents in the cuticular and internal lipids extracted from *Sarcophaga argyrostoma* [µg/g of body mass ± SD].

Sterols	Larvae		Pupae		Adults	
	Cuticular	Internal	Cuticular	Internal	Cuticular	Internal
Glycerol	3.064 ± 0.109^A^	30.369 ± 0.517^A–C^	0.316 ± 0.021^B^	0.202 ± 0.014^C^	25.366 ± 0.632^A–C^	93.437 ± 2.506^A–C^
Cholesterol	4.527 ± 0.038^A^	2.958 ± 0.015^A^	7.604 ± 0.174^A^	5.676 ± 0.196^A^	33.256 ± 0.514^A^	92.327 ± 0.509^A^

*FFA* free fatty acids, *SD* standard deviation, *ND* not detected; statistically significant differences are marked with the same letters (ANOVA, Test HSD Tukey, p < 0.05), see Table S1 for raw data.

The highest concentration of cholesterol was observed in the extracts from adult cuticular (33.256 ± 0.514 μg/g insect body mass) and internal extracts (92.327 ± 0.509 μg/g insect body mass); the highest content was observed inside the insect body. In contrast, in the larval and pupal extracts, a higher content of cholesterol was observed in the cuticular fractions of larval (4.527 ± 0.000 μg/g of insect body) and pupal extracts (7.604 ± 0.174 μg/g of insect body) than in their corresponding internal fractions: 2.958 ± 0.015 μg/g insect body mass in larvae and 5.676 ± 0.196 μg/g insect body mass in pupae. The results have been showed in Table 3.

## Discussion

The FFAs comprise a huge and diverse group of lipids. Our present findings demonstrate high quantitative and qualitative FFA content diversity between the three developmental stages: larvae, pupae and imago. They also confirm the occurrence of metamorphosis-related changes in the FFA profiles in *S. argyrostoma* as observed in other insect species^37–44^.

The differences in distribution of fatty acids in the body of the insect are summarised in Table 2. The age-dependent differences in cuticular lipid content have been observed in *S. bullata* imagoes: FFAs were found to constitute 26% of all cuticular lipids in new-borns and 45% in 7-day-old flies, suggesting that lipid synthesis or transport to the cuticle surface may be incomplete in newly-emerged adults. The author also proposes that lipid transport occurs primarily through the unhardened cuticle of newly-emerged adults, and that transport is essentially complete by the time the adult cuticle is fully hardened^56^. FFAs are also used as precursors of cuticular hydrocarbons. Oenocyte-directed RNAi knock-down of *D. melanogaster* CYP4G1 or NADPH-cytochrome P450 reductase results not only in flies deficient in cuticular hydrocarbons, but also in the accumulation of midchain fatty acids, which might suggest that they play a role in hydrocarbon synthesis^57^.

The results of the GC–MS analysis indicate that the extracts from adults possess higher FFA content than in preimaginal stages of flies, which is in accordance with previous findings^26^ in *Sarcophaga carnaria*. Sun and Brookes also report a lower level of FFA in the fat body of *Sarcophaga bullata* larvae (from 0.93 to 2.92%, depending on age) than in adults^58^; they also note that C18:1 > C16:0 > C18:0 > C16:1 FFAs predominated in three-day-old larvae, and C18:1 > C16:0 > C16:1:C18:2 in nine-day-old larvae, and that the amount of C18:2 and C16:1 increased, and C18:0 decreased, during six days of rearing^58^.

In the present study, C18:1 > C16:1 > C16:0 > C18:0 predominate in both cuticular and internal extracts from larvae and adults, while 18:1 > C16:0 > C16:1 > C18:0 predominate in pupae; those FFAs have also found to be characteristic of *Diptera*^59,60^. The dominant FFA is C18:1 in all extracts, with the highest proportions being found in the adult extracts (47.78% in cuticular and 49.14% in internal) and the internal extracts of pupae (47.85%). In previous studies, higher proportions of C18:1 have been described in the internal extracts from larvae (55.9%) and pupae (58.9%) of *S. carnaria*^26^. In *S. bullata*, lower levels of C18:1 were found in the cuticular extracts of adults; in addition, the C18:1 levels in the cuticular extract increased during adulthood from 29.0% in new-born and 34.3% in seven-day-old adults, while C16:1 decreased from 32.5 to 21.6%^56^.

In all extracts, the predominant FFA was found to be C18:1. One example of a C18:1 FFA is oleic acid. It has many biological properties, including the ability to provide a wide temperature window for growth. It provides the best environment for critical membrane proteins such as membrane ATPases, which function at optimum levels when oleic acid is present in the cell membrane^61^; therefore, an increase in oleic acid level in response to, or in preparation for low temperatures, may maintain correct fluidity of the membrane without sacrificing the delicate balance needed to optimize the function of sensitive membrane proteins. Higher proportions of C18:1 have been found as an adaptation to low temperatures in *Eurosta solidaginis*^62^, *Dolycoris baccarum* and *Piezodorus lituratus*^63^ and *S. similis*^64^. As oleic acid is energetically more favourable to manufacture than linoleic acid, due to it having one less double bond, insects that upregulate oleic acid rather than linoleic acid for low temperature use may be preserving finite energy reserves while still gaining the benefit of a wide window of fluidity^65^. The high amount of C18:1 observed in *S. argyrostoma* might be an example of adaptation to cold.

Polyunsaturated fatty acids (PUFA) are usually associated with biomembranes as phospholipid fatty acids. The proportion and composition of 20:5, 20:4 and 20:3 in the membranes, cuticle and so on vary according to life stage and tissue type^66^. Higher concentrations of C20:5 (1.59% of FFA content) and C20:4 (1.12%) were observed in the internal extracts in the present study; they are thought to be precursors of prostaglandins, leukotrienes and thromboxanes^67–70^. Various metabolites of C20:4 (arachidonic acid), known as eicosanoids, stimulate oviposition in crickets, regulate the function of Malpighian tubules in mosquitoes or ants, and control thermoregulation in cicadas^71^. They also play crucial roles in the mediation of insect cellular and humoral immunity^72,73^; for example, in *S. argyrostoma*, they were found to mediate and coordinate the biosynthesis of NO and lysozyme in response to bacterial challenge^27^, and to participate in the LPS-dependent activation of the IMD pathway in *Sarcophaga peregrina*^74^. In a study of the internal extract of *S. carnaria*, Gołębiowski and co-workers found C20:5 to be present at 90-times higher concentrations in females than males; they propose that the compounds may play an important role in vitellogenesis^26^. Clements and co-workers^75^ propose that arachidonic acid may play a role in the resistance of the Colorado potato beetle, *Leptinotarsa decemlineata* to neonicotinoid insecticide, and suggest that this may be associated with its regulatory role in cytochrome P450-dependent insecticide detoxification pathways. In the present study, a high concentration of C20:4 was observed in the extracts from the adults, which might suggest that *S. argyrostoma* is resistant to chemical insecticides; however, more detailed research is needed to confirm this.

The FFAs in the insect cuticle have also been identified as resistance factors against fungal infection; for example, Gołębiowski and co-workers report that cuticular FFAs play a role in resistance to fungal infection by the flies *Calliphora vicina*, *Lucilia sericata*, *C. vomitoria* and *S. carnaria*^26,39–41,76,77^. Also, literature data postulate that the chemical composition of cuticular FFAs may influence the susceptibility of cockroaches (*Blatella germanica, Blatta orientalis)* to infection by the fungus *Metarhizium anisopliae*^78^ and by *Conidiobolus coronatus*^79^. Additionally, Smith and Grula indicate that cuticular FFAs in corn earworm larvae (*Heliothis zea*) can inhibit the germination and growth of *Beauveria bassiana*^80^*.* These examples illustrate the significant role played by cuticular FFAs in resistance or susceptibility to entomopathogens.

In the present work, higher levels of C9:0, C18:0 and C22:0 were found in the cuticular fractions of larvae, pupae and adults than the internal fractions. Of these, C9:0 and C18:0 have been found to inhibit the germination of *C. coronatus* spores^81^. Wrońska and co-workers^43^ report a correlation between the concentration of C9:0 and C18:0 in the cuticle of *Galleria mellonella* larvae, pupae and imagoes, and the activity of *C. coronatus* enzyme cocktail. In addition, Boguś and co-workers^82^ report a correlation between the concentration of C9:0, C18:0 and C22:0 in the cuticle of four medical and veterinary important flies: *C. vomitoria, C. vicina, L. sericata* and *Musca domestica*, and the enzymatic activity of *C. coronatus*.

Lipid accumulation and mobilisation is particularly important for the radical reconstruction of body structure and its biochemistry in holometabolous insects such as the *Sarcophagae*. However, these flies can also be larviparous, meaning that the egg develops internally, and females then give birth to first-instar larvae^83^.

The lipid content of holometabolous insects increases steadily during larval development, reflecting not only the metabolic requirements of the larva, but also the need to accumulate reserves for maintenance during metamorphosis^84^. However, in the present study, a higher concentration of particular FFAs was observed in the cuticular fraction; the levels of C9:0, C17:0, C18:0 and C23:0 were twice as high, C22:0, C24:0 and C26:0 were three times as high and C25:0 was five times as high. It is unusual to find odd-numbered fatty acids in insects, and as such, the presence C25:0 and C23:0 in the larval extracts merits further discussion. Pentacosanoic acid methyl ester is used as a pheromone by the European paper wasp *Polistes dominulus* for nest discrimination^85^. The C25:0 fatty acid has been found in both larval-larval (0.3% of FFA content) and larval-pupal (0.1% of FFA content) cuticular extracts from *Dendrolimus pini* exuviae; in contrast, C23:0 has only been found in in larval-larval extracts (0.1% of FFA content)^86^. C23:0 has also been described in the whole-body extracts of *Allomyrina dichotoma*^87^*, Protaetia brevitarsis*^88^, *Tenebrio molitor*^89^ and in *Cirina forda*^90^ larvae and *Teleogryllus emma* adults^89^, which are used as food in Asia.

Cholesterol was also found in higher levels in the cuticular extracts of the tested larvae. This contrasts with *C. vicina, M. domestica* and *S. carnaria*, where higher levels have been recorded in the internal extract. In addition, in contrast to the present study on *S. argyrostoma,* it has previously been found to be absent from cuticular extracts of *S. carnaria* larvae^38^. In the present study, glycerol was found to be present at higher concentrations in the internal extract, which is similar the distribution of glycerol in *M. domestica* larvae^38^.

During metamorphosis, most larval tissues decompose and adult structures are synthesized de novo from imaginal discs^91–93^. This process is dependent upon energy reserves, lipids for example, and anabolic precursors accumulate during larval growth^84^. Our present findings indicate a wide diversity of FFAs in the pupal stage; however, lower total FFA content was observed in both the internal and cuticular extracts of the pupal stage, compared with other developmental stages. A number of FFAs were found to be present at higher concentrations in the cuticular extract than the internal one, particularly C9:0, C12:0, C14:0, C18:0 and C22:0, each of which was present at twice the level in the cuticle. Wrońska and co-workers^43^ report a high positive correlation between the concentration of C12:0 in the pupal cuticle of *G. mellonella* and the efficiency of entomopathogenic fungus *C.* *coronatus* chitinases and lipases in degrading it. In addition, similar to the present findings, *S. carnaria* pupae demonstrated a lack of C11:0, C19:1 and C20:4 in cuticular extracts and C20:1 in internal extracts; however, all FFAs were present in extracts from the larvae^26^.

In addition, the concentration of cholesterol was higher in the cuticular extracts in the present study, in contrast to extracts from *M. domestica, S. carnaria* and *C. vicina* pupae^38^*.* Glycerol was found to be present at very similar concentrations in the cuticular and internal extracts of the tested pupae, as previously observed in extracts from *C. vicina* pupae^38^. Lower amounts of internal FFAs were recorded, which might be due to disintegration of larval fat body in the pupal stage^94^.

An interesting finding was the presence of the long-chain FFA C28:0 on the surface of the cuticle of pupae, which is quite unique for insects. It is an aliphatic primary acid which has been shown to be an antibiofilm and anti-adherence agent against *Streptococcus mutans*^95^; it has so far only been detected in the cuticular wax of the honey bee *Apis mellifera*^96^ and in the cuticular fraction from larvae and pupae of *D. pini*^86^. It is important to note that this FFA is absent in extracts from species which are also consider as significant tools in forensics, such as *C. vicina*^97^*, C. vomitoria*^40^ and *S. carnaria*^26^. However, C28:0 has been found in chloroform extracts from *C. vicina* (*C. erythrocephala*) pupae by electron diffraction^98^*.*

The advantage of holometabolous development is the specialisation of stages: larvae for feeding and growth, and adults for reproduction. However, there are some examples of holometabolous insects, such as blood-feeding mosquitos, which require blood meal to obtain protein or lipids to achieve, or enhance, reproductive success. Research on *S. crassipalpis* has shown that lipids derived from adult dietary components constitute half of the storage materials of eggs, and these are used as an energy supply for the developing embryos^99^. The balance between lipogenesis and lipolysis is tightly regulated in insects, to match energy needs that vary in response to the changes in the environment^100–102^. FFAs are main source of energy for muscles during flight.

In the adult fly extracts, higher concentrations of particular short-FFA were observed in the cuticle than in the internal extracts; for example, the concentration of C5:0 was four-times higher in the cuticle. However, the opposite was observed in case of middle and long-chain FFAs: higher amounts of particular FFA was detected inside the body, particularly C20:4 and C20:5. Also, the cholesterol and glycerol concentrations were higher in the internal extracts; a similar situation was observed in extracts from *M. domestica, S. carnaria* and *C. vicina* flies; however, higher concentrations were observed in the cuticular fraction for male *C. vicina*^38^.

The internal extract also included two unsaturated FFAs: C12:1 (0.02% of all FFAs) and C18:3 (0.37% of all FFAs). C12:1 has been found in extracts from the larvae, pupae and adults (both female and male) from two species: *Dermestes ater* and *Dermestes maculatus*, which are highly resistant to infection by the entomopathogen fungus *C. coronatus*^42^, as well as in cuticular extracts from *Schistocerca gregaria*^103^. The presence of C18:3 was detected in phospholipids in *Sarcophaga similis*^64^ and in extracts from *Culex pipiens* mosquito, where it allowed adults to stand or hop on the medium surface^104^ and support flight at emergence; however, it was less effective than arachidonic acid (C20:4)^105^. C18:3 is required by Lepidoptera and Hymenoptera to achieve complete metamorphosis^106^ and has been used as a precursor of female moth sex pheromones^107^.

The fatty acid contents of insects can vary according to growth stage, temperature and dietary regime. It is well know that in the environment, flesh flies feed on nectar, fruit juice, decomposing matter such as excrement, and carrion as sources of protein^108–110^. Valverde-Castro and co-workers report the presence of high population densities of *Sarcopagiae* flies in places with decomposing fish, whose flesh is associated with high fat and protein content; both are needed by female flies for developing eggs, and for the growth and development of first instar larvae^5^. Insects from fly families like the Muscidae, Calliphoridae, Drosophilidae, and Stratiomyidae can develop on media containing human faeces and fruits; however, the authors note that the nutrient levels from that source are insufficient for the larval development of the Sarcophaginae^5^. In the present work, all adults had access to both meat, i.e. beef, and sugar. Studies have shown that more than 30% of the fatty acid content in beef is composed of oleic acid^111–113^, which might explain the high level of C18:1 in the extracts from adult flies. In addition, beef is a popular meat used for feeding Diptera flies, and high levels of C18:1 have also been observed in extracts from adult *L. sericata*^39^ or *C. vomitoria*^40^. The higher levels of FFA in the extracts from adults might be explained also by the conversion of saccharose (sugar) to lipids^114^. Literature data indicates that female *Sarcophaga* start to feed on sugar after emergence and begin to feed on meat after three days, and that meat feeding is cyclic^115^. The insects are also able to take up dietary C20:5, which can alter the overall FFA profile of tissue phospholipids in lengthy feeding experiments^116–118^; in addition, dietary administration of C20:5 might reverse the inhibitory effect of dexamethasone (an inhibitor of eicosanoid formation) during viral infection in the larvae of the parasitic wasp *Pimpla turionellae*^119^.

Although sterols are essential substrates for insect steroid hormone (ecdysteroid) synthesis^120^, insects do not have the ability to synthesize sterols de novo due to a deficiency of the necessary enzymes^121,122^; therefore, their diet is their main source. Research has indicated that some insects demonstrate a preference toward some FFAs in the diet. For example, the adult mosquitoes *Aedes aegypti*^123^ and *Anopheles gambi*^124^, and the nymphal bug *Triatoma infestans*^125^ are attracted by specific FFAs (mixed with L-lactic acid), and that some FFAs might discourage insects, like flies^126^ or mosquitoes^127^. However, this preference for some FFAs could change over the course of development; for example, *Drosophila melanogaster* larvae prefer unsaturated FFAs whereas adults prefer saturated FFAs^128^.

Although diet generally affects the fatty acid profiles of insects, exceptional cases can occur. The biosynthesis of saturated palmitic (C16:0) and stearic acids (C18:0) and monounsaturated oleic acid (C18:1) seems to be widespread among insects, and accordingly, these fatty acids are the most abundant in their bodies^40,46^. Some insects from the Hymenoptera (*N. vitripennis*, for example) can synthesize the C18:2 FFA linoleic acid from oleic acid, thanks to presence of D12-desaturase, an enzyme, which is responsible for inserting a double bound at the D12-position^129^.

One of the important roles of health officials, particularly in tropical and subtropical regions, is the control of fly populations in urban and rural communities. Parasitoid wasps in the Order Hymenoptera (for example *N. vitripennis*) are regarded as biological control agents of flies^8,130–133^. *N. vitripennis* wasps have been found to demonstrate a strong preference for *Sarcophaga* pupae, including *S. argyrostoma,* due to the greater production and rapid development of wasp pathogeny during infection by^6,134,135^.

The host preference might be connected with lipid metabolism: as parasitoid wasps are supposed to have lost their potential for lipogenesis during evolution due to environmental compensation, host lipids may well be essential and limiting factors for developing larvae^136,137^. In addition, Thompson and Barlow propose that the level of FFAs in parasitic Hymenoptera is determined by the FFA levels in their host lipid profile^138^. Similarly, research on parasitized *S. bullata* or *S. crassipalpis* pupae have shown increasing expression of genes involved in lipid biosynthesis and higher lipid content after venom injection^134,139–142^. Hyperlipidaemia is also observed after administration of venom of *Euplectrus separate* to lepidopteran larva *Pseudaletia separate*^143^*.*

Our present findings illustrate the great diversity of FFAs between insect development stages: C23:0 and C25:0 are only present in larvae, C28:0 in the pupal cuticle, and C12:1 and C18:3 in the internal extracts from adults. This variation, occurring as a result of the remodelling processes of holometabolic insects and their adaptation to environment conditions might be a useful tool for the distinction of sarcophoids. Our findings regarding the chemical composition of the fatty acids of *S. argyrostoma* may play an important role in further studies on other significant lipid components of flies intended to improve our understanding of the taxonomy and physiology of insects, and thus may have a great impact on medical, veterinary, toxicology and forensic science.

## Methods

### Insect

*S. argyrostoma* were reared at 25 °C with 70% relative humidity and a 15:9 h photoperiod. The larvae were fed on beef ad libitum. The flies formed a puparium 14 days after hatching, and the adults emerged 14 days later. The species was confirmed by prof. Krzysztof Szpila from the Chair of Ecology and Biogeography (Nicolaus Copernicus University in Toruń, Poland). Post-feeding third instar larvae, freshly emerged pupae and 6-day-old sexually mature adults were used for lipid extraction. The sixth generation of insects were used in the study.

### Extraction of free fatty acids (FFAs)

Cuticular and internal lipid components of insects were extracted, separated and analysed by GC–MS. The method of extraction was based on literature data (for example^43,44,76,79,144^). Lipids from larvae, pupae and adults were extracted first in 20 ml of petroleum ether (Merck Millipore, Germany) for 5 min (extract I) and then again in 20 ml of dichloromethane (Merck Millipore, Germany) for 5 min (extract II) to yield cuticular lipids. The insects were sonicated (1 min) with dichloromethane to produce Extract III containing internal lipids. The extracts were placed in glass flasks and evaporated under nitrogen.

### Derivatization method

One mg of each sample and 10 µl 19-methylarachidic acid (1 mg/ml; Merck Millipore) were silylated with 100 μl of N,O-Bis(trimethylsilyl)trifluoroacetamide (BSTFA): chlorotrimethylsilane (TMCS) (99:1) (Merck Millipore) mixture for one hour at 100 ◦C to obtain trimethylsilyl esters (TMS) of FFAs. The TMS values of the fatty acids were then analysed by GC–MS.

### GC–MS analyses

The GC–MS analyses were carried out on a GCMS-QP2010 system with mass detector (Shimadzu, Japan). Helium was used as the carrier gas at a column head pressure of 65.2 kPa. A DB-5 MS (Zebron, Phenomenex, USA) column was used (thickness 0.25 µm, length 30 m, diameter 0.25 µm). The column oven temperature cycle was maintained at 80 °C for 3 min, then ramped from 80 to 310 °C at 4 °C/min; the final temperature was then held for 10 min. The ion source temperature was 200 °C and the interface temperature was 310 °C. Split mode was used with a split ratio of 10. All compounds were identified based on fragmentation patterns and mass-to-charge ions of the TMS derivatives and the NIST 11 library. The mass spectra of the fatty acid trimethylsilyl esters comprised M + (molecular ion), [M-15] + , and fragment ions at m/z 117, 129, 132, and 145. GC analysis used 19-methylarachidic acid (1 mg/ml; Merck, Germany) as an internal standard (IS). The content of the compounds in the analyzed samples was calculated from the chromatogram peak areas. The results were expressed as a means standard deviation of three GC/MS analyse. Response factors of one were assumed for all constituents. The method is based on literature data^43,44,76,79,144^.

### Statistics

The findings were tested by the one-way analysis of variance (ANOVA). Tukey’s test was used for post hoc analysis. Each test was performed separately. All analyses were performed using Statistica 6 software (StatSoft Polska, Poland). Differences were significant at p < 0.05.

## Supplementary information

Supplementary Legend.

Supplementary Table S1.
