# Supplementary material for: Metamorphosis-related changes in the free fatty acid profiles of Sarcophaga (Liopygia) argyrostoma (Robineau-Desvoidy, 1830)
Source: Sci Rep. 2020 Oct 15;10:17337. doi: 10.1038/s41598-020-74475-1 (PMC7562915; doi:10.1038/s41598-020-74475-1)
Supplement: Supplementary file 1 — Supplementary Legend. [file 41598_2020_74475_MOESM1_ESM.docx]

# Supplementary information

**Table S1** - A comparison of the FFA profiles and glycerol and cholesterol content of the cuticle surface (sum of extracts I and II) and the internal structures of *S. argyrostoma* – the raw data.
